# Severe Hyponatremia Precipitated by Acute Urinary Retention in a Patient with Psychogenic Polydipsia

**DOI:** 10.1155/2020/8792897

**Published:** 2020-07-30

**Authors:** C. M. H. Hilton, L. Boesby, K. E. Nelveg-Kristensen

**Affiliations:** ^1^Department of Cardiology, Nephrology and Endocrinology, Nordsjællands Hospital-Hillerød, 3400 Hillerød, Denmark; ^2^Department of Otorhinolaryngology, Nordsjællands Hospital-Hillerød, 3400 Hillerød, Denmark

## Abstract

A woman in her late sixties presented with severe hyponatremia and acute kidney injury (AKI) as consequence of psychogenic polydipsia and acute urinary retention due to urinary tract infection. Urinary catheterization promptly drained 5.5 L of urine with resulting polyuria, leading to an initial swift raise in plasma (P) sodium concentration, disregarding the course of fluid resuscitation. After the polyuric phase, normal range P-sodium levels were reestablished by oral water restriction. Treatment with psychoactive drugs, e.g., zuclopentixol, may have contributed to the severity of the condition. There are few published reports regarding water intoxication and urinary retention, but none reflecting severe hyponatremia precipitated by acute urinary retention in a patient with polydipsia. By this report, we illustrate the detrimental consequences on water and electrolyte homeostasis of urinary retention and polydipsia resulting in acute water intoxication. The purpose of presenting this case is firstly to draw attention to the potentially fatal combination of polydipsia and postrenal acute kidney injury, where the kidneys are unable to correct the enormous excess water, then to focus on the difficulty in correcting hypervolemic hyponatraemia in the context of polyuria after relief of urinary retention, and finally, to point out that patients in treatment with antipsychotics may have further worsening of electrolyte derangement.

## 1. Introduction

Hyponatremia is a frequently encountered electrolyte abnormality [1]. Of all hospitalized patients, 14–38% develop hyponatremia during hospital stay, [2, 3] and approximately 15% of all patients acutely admitted to hospital have a P-sodium < 135 mmol/L [4]. Hyponatremia is associated with a marked increase in mortality and morbidity resulting from, e.g., cerebral oedema and central pontine myelinolysis. Even mild cases are independently associated with a higher risk of in-hospital and long-term mortality [5, 6]. Psychogenic water intoxication is a well-known cause of hyponatremia and, hence, plasma hypo-osmolality [7]. People with normal kidney function are protected from developing severe electrolyte derangements by effective renal compensation. Impaired renal function, as a consequence to postrenal obstruction, potentiates the harmful effects of excessive water intake.

Hyponatremia in acutely admitted medical patients is often multifactorial. Causes are Syndrome of Inappropriate Antidiuretic Hormone Secretion (SIADH) (22%), heart-, liver- or kidney-insufficiency (23%), thiazides (20%), correction of fluid losses with hypotonic solutions (17%), loop-diuretics (8%), and others (10%) [8]. In-hospital acquired hyponatremia is primarily caused by hypotonic fluid therapy. Hypo-, hyper-, and normovolemic hyponatremia should be treated differently.

## 2. Case Presentation

A woman in her late sixties with schizoaffective disorder, managed with zuclopenthixol and oxazepam, was transferred from the psychiatric department to the acute care facility with polydipsia, urinary retention, and hyponatremia. She had no history of hyponatremia, polydipsia, or urinary retention and was not treated with diuretics. The laboratory charts showed P-sodium within the normal range of 136–142 mmol/l and a normal kidney function (P-creatinine 55–70 *μ*mol/l).

**Figure 1:**
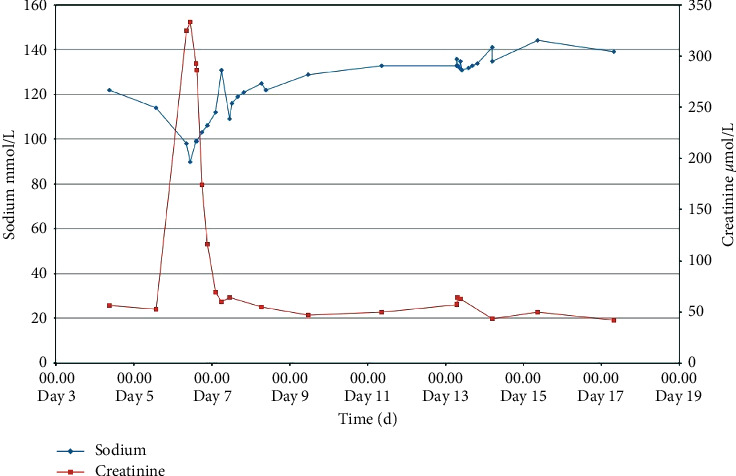
Blood samples reflecting P-sodium (mmol/L) and P-creatinine (*μ*mol/L) levels over days.

The patient was initially admitted to the psychiatric ward in a psychotic condition with a Glasgow Coma Scale (GCS) of 14-15 and polydipsia, suspected of not having taken her antipsychotic medication. The excessive water intake was handled by fluid restriction, which was not fully followed. P-sodium was not initially measured. Four days after admission, the P-sodium was 122 mmol/L (137–145 mmol/L), and at day six, after several episodes of vomiting, the P-sodium concentration had decreased to 98 mmol/L (Figure 1). The patient was transferred to the acute care facility, presenting with GCS 10 and diffuse abdominal soreness. A urinary catheter was placed, and 5.5 L of red coloured urine was emptied immediately. Fluid therapy with 0.9% saline at an infusion rate of 50 ml/hour (h), daily weighing, fluid input/output registration, blood samples every 3-4 hours, and a 24-h urine sample were initiated. During the next nine hours, the patient became increasingly polyuric with a total diuresis of 5.5 L. Simultaneously, after an additional decrease to 90 mmol/L, P-sodium rose to 112 mmol/L, after which the saline infusion was removed. Antibiotics were given empirically due to leucocytosis, elevated C-reactive protein, and signs of urinary tract infection. After these initial days of fluctuation, the P-sodium slowly rose, primarily due to water restriction.

## 3. Investigations

Blood samples obtained in the acute care facility showed a P-osmolality of 216 mosmol/kg (280–290 mosmol/kg), P-creatinine 333 *μ*mol/L (45–90 *μ*mol/L), and P-potassium 6.0 mmol/L (3.5–4.4 mmol/L). Urine- (U-) osmolality was 235 mosmol/kg (>560 mosmol/kg), and U-sodium was 83 mmol/L (20–40 mmol/L).

Nine hours from admission, the P-sodium had risen to 112 mmol/L, and after another four hours, it further rose to 131 mmol/L (Figure 1). P-sodium returned to the normal range after eight days. Hyperkalemia was corrected as the renal function returned to normal 12–15 hours after urinary catheterization.

An abdominal computed tomography (CT) imaging described a flaccid, slightly thickened urinary bladder and a uterus fibroma, discreet free abdominal fluid, slight hydroureteres bilaterally, discreet unilateral hydronephrosis, and discreet pleural effusion, predominantly on the right side.

## 4. Outcome and Follow-Up

One week after admission to the acute care facility the patient had two general tonic clonic seizures lasting more than two minutes. There was no lateralization upon neurological examination. The patient was treated with diazepam, fentanyl, propofol, a loading dose of fosphenytoin and was intubated. Thereafter, she was treated with tinzaparin, lamotrigine, and valproate. The patient had no history of epilepsy, seizures, or any other neurologic disease. A cerebral CT scan and magnetic resonance imaging (MRI) showed no focal cerebral damage, specifically no pontine signal changes, and it was concluded that the seizures should be explained by vast electrolyte fluctuation and treatment with zuclopenthixol, which lowers the convulsive threshold.

## 5. Discussion

By the present case report on a patient with psychogenic water intoxication, we demonstrate the detrimental consequences of acute urinary retention and AKI, resulting in impaired renal fluid and electrolyte regulation. This was indeed emphasized by the abrupt fall in P-sodium, which initially was forced by the inability of free-water clearance as the urinary tract was obstructed. Furthermore, after urinary catheterization, the relatively high U-osmolality to P-osmolality of 1.09, which under normal renal conditions should have been much lower in the setting of acute water intoxication [9], is most reasonably explained by renal tubule damage as a consequence to severe urinary retention, resulting in an inability of adequate sodium reabsorption [10]. This was further stressed by a relatively long resuscitation period, before normal-range P-sodium was achieved. Water intoxication with resulting hyponatremia and hypoosmolality is a well-known challenge among patients with psychiatric disorders [7, 8]. In addition to the massive water overload, hyponatremia itself, in patients with psychiatric diseases, has been associated with resetting of the hypothalamic osmoreceptors, resulting in secretion of ADH (Anti Diuretic Hormone) at a lower P-osmolality [11]. Effective fluid restriction was not carried out prior to acute care facility admission, as indicated by a drop in the P-sodium from 122 to 98 mmol/l.

A timely differentiation between acutely and chronically developed hyponatremia of 48 hours has been widely accepted. Nevertheless contemporary international guidelines recommend that severe symptomatic hyponatremia should be treated promptly with 100 ml 3% saline administered intravenously over 10 minutes and that this procedure may be repeated up to three times, since the risk of acute cerebral oedema far exceeds the risk of osmotic demyelination [12]. Subsequent correction of the condition should proceed with the administration of 0.9% saline intravenously (IV) at an infusion rate of 50 ml/h. Furthermore, guidelines recommend a maximal change in the P-sodium of 8 mmol/L over 24 hours and no more than 1 mmol/L/h in acute hyponatremia and 0.5 mmol/L/h in chronic conditions. If correction has proceeded more rapidly than desired, the risk of osmotic demyelination may be reduced by IV administration of 5% glucose, 0.45% NaCl, or desmopressin. In the current case report, our patient was initially treated with 50 ml 0.9% saline/h; however, one can argue that a bolus of 3% saline should have been initiated as she presented with a GCS of 10 and other neurologic symptoms. Interpretation of the neurologic evaluation was hampered by her psychotic condition. Moreover, as the patient became polyuric, putatively with a rapid rise in free-water clearance, administration of hypertonic saline could cause a fatal increase in P-sodium concentration. It is difficult to predict the tubular function in a patient with AKI; however, in this case, where the hyponatremia was induced by severe fluid overload, expanding the ECV with IV isotonic saline did not increase p-sodium. The subsequent fall in s-sodium could be explained by, e.g., residual water absorption from the gut or excessive tubular sodium excretion coincided by fluid retention due to an increased ADH response, which would be in keeping with the initial high U-sodium concentration [13].

The kidneys possess counteracting mechanisms that allow for urinary concentration and dilution. With normal renal function, the total solute concentration of body fluids remains constant, despite fluctuations in solute and water intake. During excessive water intake, the renal free-water clearance may produce up to 25 L of urine per day; however, in cases of severe water deprivation, the daily urine production can be reduced to less than 0.5 L [12]. In incidents of impaired kidney function, as in the current case with postobstructive AKI, the harmful effects of polydipsia increase.

Although our patient was unable to adequately dilute her urine, the subsequent polyuric phase may have contributed to the fast initial rise in P-sodium (Figure 1). We interpret the outlying blood sample with a P-sodium of 131 mmol/L as a measurement error, as P-sodium before and after this measurement, with a four-hour margin between each blood sample, was 112 mmol/L and 109 mmol/L, respectively. Fluctuations in the P-sodium of 18–21 mmol/L within four hours in the current case are most unlikely to have occurred.

Urinary tract obstruction per se can lead to hyponatremia. A patient undergoing urea diuresis after relief of urinary tract obstruction will lose sodium and water leading to hypovolemia and hyponatremia [12]. This patient's drastic rise and fall in P-creatinine, before and after urinary catheterization, respectively, suggests acute urinary obstruction as a contributing factor to AKI, although there probably pre-existed some level of chronic urinary retention. In this regard, a previous report showed that correction of chronic urinary obstruction reduced the incidence of hyponatremia in a polydipsic patient, [14], and urinary retention and polyuria are both registered as common (1–10%) side effects to zuclopenthixol, with which this patient was treated upon admission. Nonetheless, these considerations taken into account, we believe that urinary tract infection was the primary cause of acute total obstruction in the current case [15].

Collectively, the combination of polydipsia, urinary retention, and postobstructive AKI holds a serious risk of severe hyponatremia and hypoosmolality, with subsequent increased morbidity and risk of fatal outcomes. Furthermore, in the setting of acute water intoxication, postobstructive polyuria and sodium loss due to postobstructive tubular damage may contribute to a less predictable and prolonged resuscitation period [16]. Physicians should be aware of these mechanisms, especially among patients with psychogenic polydipsia treated with psychoactive drugs.

## 6. Learning Points/Take-Home Messages

- Be aware of urinary retention in patients with water intoxication
- Follow sodium levels more frequently after relieving urinary retention in a patient with hyponatremia
- Patients with urinary retention are not able to adequately regulate sodium levels
- Possibly, urinary retention can cause a picture similar to SIADH
- Possibly, urinary retention should be relieved more gently

## References

[1] Holm E. A., Brorson S. W., Kruse J. S., Faber J. O., Jespersen B. (2004). Hyponatremia in acutely admitted medical patients--occurrence and causes. *Ugeskr Laeger*.

[2] Chung H.-M., Kluge R., Schrier R. W., Anderson R. J. (1987). Clinical assessment of extracellular fluid volume in hyponatremia. *The American Journal of Medicine*.

[3] Fenske W., Maier S. K. G., Blechschmidt A., Allolio B., Störk S. (2010). Utility and limitations of the traditional diagnostic approach to hyponatremia: a diagnostic study. *The American Journal of Medicine*.

[4] Holland-Bill L., Christiansen C. F., Heide-Jorgensen U. (2015). Hyponatremia and mortality risk: a Danish cohort study of 279508 acutely hospitalized patients. *European Journal of Endocrinology*.

[5] Waikar S. S., Mount D. B., Curhan G. C. (2009). Mortality after hospitalization with mild, moderate, and severe hyponatremia. *The American Journal of Medicine*.

[6] Giuliani C., Peri A. (2014). Effects of hyponatremia on the brain. *Journal of Clinical Medicine*.

[7] Gebel F., Meng H., Michot F., Truniger B. (1989). Psychogenic water intoxication. *Schweiz Med Wochenschr*.

[8] Berl T., Schrier R. W., Schrier R. W. (2010). Disorders of water metabolism. *Renal and Electrolyte Disorders*.

[9] Armstrong L. E., Johnson E. C., Munoz C. X. (2013). Evaluation of Uosm:Posm ratio as a hydration biomarker in free-living, healthy young women. *European Journal of Clinical Nutrition*.

[10] Klahr S., Harris K., Purkerson M. L. (1988). Effects of obstruction on renal functions. *Pediatric Nephrology*.

[11] Goldman M. B., Robertson G. L., Luchins D. J., Hedeker D., Pandey G. N. (1997). Psychotic exacerbations and enhanced vasopressin secretion in schizophrenic patients with hyponatremia and polydipsia. *Archives of General Psychiatry*.

[12] Parikh C., Berl T. (2010). Disorders of water metabolism. *Comprehensive Clinical Nephrology*.

[13] Steele A., Gowrishankar M., Abrahamson S., Mazer C. D., Feldman R. D., Halperin M. L. (1997). Postoperative hyponatremia despite near-isotonic saline infusion: a phenomenon of desalination. *Annals of Internal Medicine*.

[14] Itoh N., Fuwano S., Matsui N., Takagi R. (1997). Reduction of hyponatremia in a schizophrenic with polydipsia-hyponatremia syndrome by surgical intervention. *Psychiatry Research*.

[15] Selius B. A., Subedi R. (2008). Urinary retention in adults: diagnosis and initial management. *American Family Physician*.

[16] Halbgewachs C., Domes T. (2015). Postobstructive diuresis: pay close attention to urinary retention. *Canadian Family Physician*.

